# Sudden death in the young adult: a Tunisian autopsy-based series

**DOI:** 10.1186/s12889-020-10012-z

**Published:** 2020-12-17

**Authors:** Said Saadi, Sami Ben Jomaa, Mariem Bel Hadj, Dorra Oualha, Nidhal Haj Salem

**Affiliations:** 1 Department of Forensic Medicine, Fattouma Bourguiba Teaching Hospital, 5000 Monastir, Tunisia; 2 Faculty of Medicine, University of Monastir, 5000 Monastir, Tunisia

**Keywords:** Legal medicine, Sudden death, Young adult, Autopsy, Retrospective studies, Sudden cardiac death

## Abstract

**Background:**

We aim to study the profile, and pathological characteristics of sudden death in young in purpose of recommendations for prevention.

**Methods:**

We performed a retrospective cohort study using autopsy data from the Department of Forensic Medicine of Monastir (Tunisia). A review of all autopsies performed for 28 years was done (August 1990 to December 2018). In each case, clinical information, and circumstances of death were obtained. A complete forensic autopsy and histological, and toxicological investigations were performed. We have included all sudden death in persons aged between 18 and 35 years.

**Results:**

We collected 137 cases of sudden death during the studied period. The mean age of the studied population was 26.47 years. Almost 72% deaths were classified as cardiac death, and was due to ischemic heart disease in 32.32%. Sudden death was attributed to a pleuropulmonary cause in 7.4%, an abdominal cause in 6%, and from a neurological origin in 4.5%. The cause of sudden death in this group was not established by 9.5%.

**Conclusion:**

In this series, sudden death in young adults occurs mainly in a smoking male, aged between 18 and 24 years old, occurring at rest, in the morning, and early in the week. It is more common, especially in summer. Sudden death is most often the first manifestation of pathologies, especially unsuspected heart diseases. The predominance of cardiovascular causes is the common denominator of almost all studies reported in the literature. Our findings suggest that prevention of sudden death among young adults under the age of 35 years should also focus on evaluation for causes not associated with structural heart disease.

## Background

Sudden death is defined as unforeseen or rapid death, of natural origin, unexpectedly occurring in a person in good apparent health [1]. In the elderly and adults, sudden death is quite common [1]. In France, for example, it represents 60,000 deaths per year [1]. Some studies have demonstrated that there is a divergence in the incidence of sudden death in young adults [2–5]. The published studies and the work carried out give an epidemiological idea about specific categories such as young adults, rarely children, and adolescents. In young adults, attention is instead given to deaths occurring in young soldiers and younger subjects during the sporting activity [2–5].

The occurrence of death in a young adult remains an event experienced as dramatic for the family as well as society and doctors. Sudden death at any age constitutes a medico-legal barrier to burial. The practice of a medico-legal autopsy is mandatory. Sudden death is less common in the age group between 18 and 35 years [6].

Our study is one of the few studies that only consider young adults, and studies all the causes of sudden death in this age group. The causes of sudden death vary from one country to another depending on the genetic characteristics of the population as well as demographic, environmental, and lifestyle characteristics.

The cause of death remains variable. However, in 6 to 40% of cases, the classic autopsy does not allow the diagnosis to be made despite the completion of complementary examinations [1]. As a result, we started this work to determine the features of sudden death in young adults in the study population, and to determine the etiologies of sudden death in the young subject.

## Methods

### Type of study

Our work is a descriptive, retrospective, and exhaustive study of 137 cases of sudden death in young adults collected at the Department of Forensic Medicine of Fattouma Bourguiba University Hospital in Monastir during a period of 28 years from August 1990 to December 2018.

### Study population

From the records of subjects who had undergone a medico-legal autopsy during the period aforementioned, there were 137 cases of sudden death of young adults aged between 18 and 35 years old.

The Inclusion criteria were:

- Young adult subject between 18 and 35 years old.
- Sudden death (unexpected, and rapid death with the absence of medical record that may explain the death).
- Natural cause of death confirmed by pathological examination, and toxicological analyses.
- Forensic autopsy done with anatomopathological and toxicological analyses.
- Obscure autopsies.

Accidental, suicidal, and criminal deaths, putrefied bodies and a body that undergone external examination only were excluded from the study.

### Data collection

The records of the Department of Forensic Medicine of the Fattouma Bourguiba University Hospital in Monastir (Tunisia) were consulted, as well as forensic records relating to cases of sudden death of young adults. Each file has 3 elements: a judicial requisition, a commemorative reminder, and the report of the forensic autopsy. These elements were our reference for the clinical information of the subject.

During the external examination, the body was described by specifying: the size, the sex, the corpulence. Based on cadaveric signs, the coroner may estimate the approximate time of death. The body has been carefully examined to eliminate traces of violence or intoxication. The autopsy itself involved a thorough examination of the organs.

The standard toxicological screening was done at each autopsy to avoid missing a toxic death. The toxicological studies were carried out at the Biochemistry, and the Toxicology Laboratory at the Fattouma Bourguiba University Hospital in Monastir. Systematic researches of the most incriminated substances in the toxic death in our country have been carried out (alcohol, benzodiazepines, barbiturates, tricyclic antidepressants, organophosphorus ester, ...) in blood, urine, and gastric contents.

An anatomopathological study has been performed systematically. It consists of tissue removal of a lesion that could be the cause of death, or from several organs if no gross abnormality was found. For all studied cases, cardiac samples were examined at the Department of Pathology in the same center. It consists of a systematic tissue sample of at least five fragments of the heart (left ventricle, septum, and right ventricle) as well as the macroscopically ischemic suspected lesion, and marked with a wire.

### Data analysis

The collected data were captured, and processed using the SPSS 22 software. An analytical study was done to study the characteristics of each cause of death. The chi-square test was applied to determine if there is a significant statistical difference between the different parameters studied. The significance rate of *P* value was set at 0.05.

## Results

### General characteristics

**Fig. 1:**
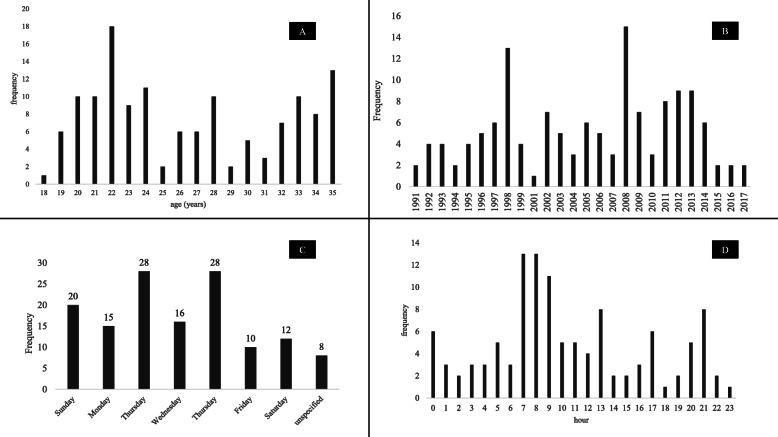
Distribution of age (**a**), year (**b**), day (**c**) and hour (**d**) of death

The population consists of 107 males (78.1%), and 30 females (21.9%) with a sex ratio (male/female) of 3.6. The average age of both sexes combined was 26.5 years (±5.3). We noted two frequency peaks: the first was at the age of 22 (13.1%), and the second at the age of 35 (9.5%) (Fig. 1a). In 71.7% of cases, these are single people. Most of the deaths occurred among day laborers (31.5%) or unemployed (28.4%).

The history of sudden death of a young subject in the family was reported in seven cases (5.1%). In these cases, no investigation was done before the death of the deceased. Personal medical history was noted in 17 cases (12.4%). It was primarily a history of cardiovascular disease (Table 1).

**Table 1:** Characteristics of the study population

Features	Number/Percentage	*P* value
Sudden death of young adult / sudden death of any age	4.3%	
Incidence of Sudden death in young adult/year	4.9	
Mean age (standard deviation)	26.5 (±5.3)	
Gender, *n* = 137		
*Female*	30 (21.9%)	
*Male*	107 (78.1%)	
*Sex ratio (m/f)*	3.6	
Medical history		
*Family history of sudden death*	7 (5.1%)	0.093
*Personal background*	17 (12.4%)	
*Smoking*	64 (46.7)	
*Unspecified*	113 (82.5%)	
Season		
*Summer*	38 (29.9%)	0.148
*Winter*	29 (22.8%)	
*Autumn*	28 (22%)	
*Spring*	24 (18.9%)	
Circumstances of death		
*At rest*	57.5%	0.293
*During sleep*	18.9%	
*Unspecified*	23.6%	
Premonitory signs		
*Faintness*	59.1%	0.115
*Others*	30.7%	
*Unspecified*	10.2%	

Smoking was reported in 64 cases (46.7%), and alcohol consumption was reported in 16 cases (11.7%). In 14 cases, we found significant chronic alcohol, and tobacco consumption. Physical activity was described in four cases including one case of professional sports practice.

The mean annual number of sudden deaths in the young adult was 4.9 cases/year. We observed two peaks in 1998, and 2008 (13 and 15 cases respectively). The number of cases has changed from 60 cases between 1991 and 2004 to more than 77 cases in subsequent years. Besides, during the 2010–2014 period (only 5 years), 35 cases were noted. However, there was no statistical significance in sudden deaths by the year with *P* = 0.238 (Fig. 1b).

Sudden death of young adults occurs more frequently in the summer (29.9%). When compared to other seasons, there is no statistically significant difference in sudden deaths by the season.

### Day of death

We found a peak frequency of sudden death in young adults on Tuesday, and Thursday (20.5% each). There was no statistically significant difference in sudden death by the day of the week with *P* = 0.522 (Fig. 1c).

### Hour of death

Two frequency peaks were observed. Death occurs more frequently at 7 am, and 8 am (Fig. 1d). The majority of sudden death cases occurred between 0 am, and 9 am with 53.4% of cases.

### Circumstances and premonitory signs of death

In our study, death occurred in 57.5% of cases at rest. Death was observed during sleep in 18.9% of cases. In 14 cases (10.2%), the clinical manifestations preceding death were not mentioned in the autopsy report. The occurrence of faintness before death was the most reported by data collection (59.1%).

### Complementary examinations

Toxicological screening of “standard” toxics, and drugs was performed on blood samples, urine, and gastric contents. In 12 cases, drug testing was positive but at a therapeutic, non-lethal threshold. These medications are tricyclic antidepressants, antiepileptic drugs or salicylates. In two cases, a non-lethal level of alcohol in the blood was found. In one case, the blood test detected the presence of amphetamines at a non-lethal level. In the rest of the cases, the analysis came back negative.

The determination of blood troponin I (cardiac and peripheral) and pericardial fluid was performed in 13 cases. In five cases, the concentration was high, reflecting an acute myocardial anomaly.

### Determined causes of death

The leading cause of sudden death in our study was cardiovascular (72.6%), followed by pleuropulmonary causes (7.4%,) and abdominal pathologies (6%). However, the etiology of death remained indeterminate in 9.5% of cases. The details concerning the main causes of death are presented in the (Table 2).

**Table 2:** Causes of sudden death in the young adult in the study population

Cause of death	Frequency *(n = 137)*
Cardiovascular	99 (72.6%)
*Myocardial ischemia*	32 (32.32%)
*Rhythmic disorder*	25 (25.25%)
*Hypertrophic cardiomyopathy*	16 (16.16%)
*Myocarditis*	9 (9.09%%)
*Vascular*	7 (7.07%)
*Valvular*	4 (4.04%)
*Intracardiac hydatid cyst*	2 (2.01%)
*Other*	4 (4.04%)
Pleural and pulmonary	10 (7.4%)
*Infection*	4
*Asthma attack*	2
*Pneumothorax*	2
*Other*	2
Abdominal	8 (6%)
*Liver hydatid cyst rupture*	2
*Peritonitis and intestinal occlusion*	2
*Pheochromocytoma*	1
*Other*	3
Neurological	6 (4.5%)
*Seizure*	2
*Cerebral haemorrhage*	2
*Other*	2
Undetermined	13 (9.5%)

**Fig. 2:**
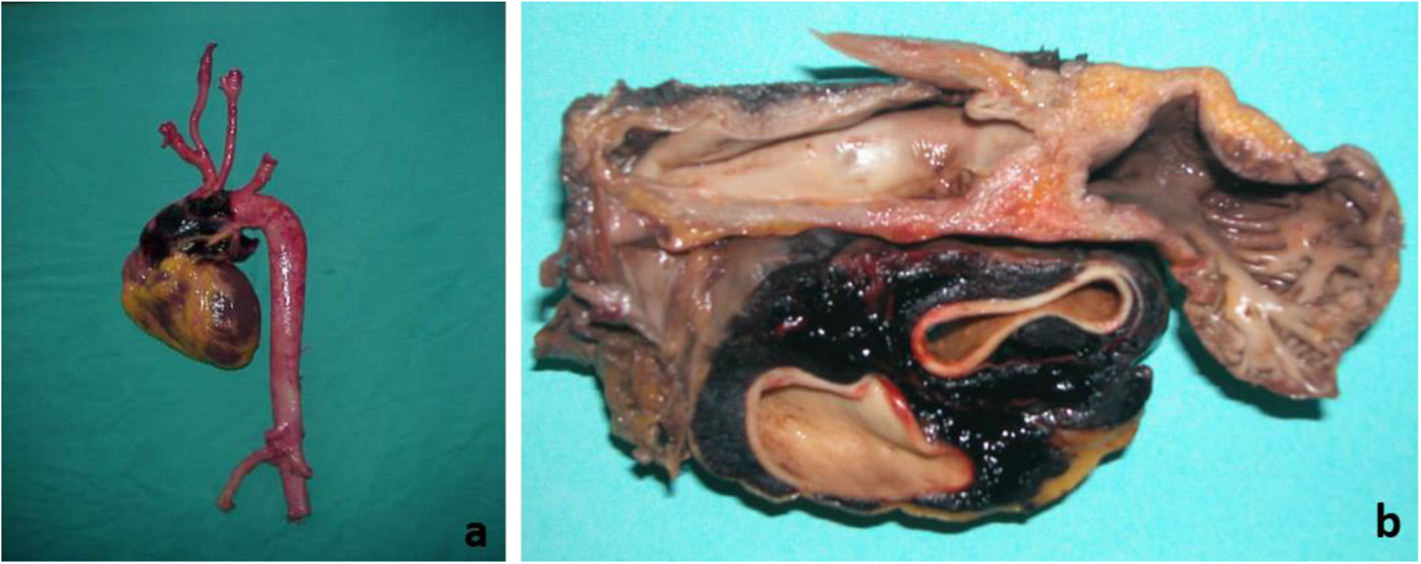
Dissection of the ascending aorta (**a**: overview, **b**: dissection)

Cardiovascular causes were mainly represented by myocardial ischemia, rhythmic disorder, hypertrophic cardiomyopathy, myocarditis, intracardiac hydatid cyst, and vascular dissection (Fig. 2), etc... (Table 2). Cardiovascular causes were more frequently responsible for death in younger age groups (ages ranging from 18 to 24 years old). This difference between ages was statistically significant (*P* = 0.001).

**Fig. 3:**
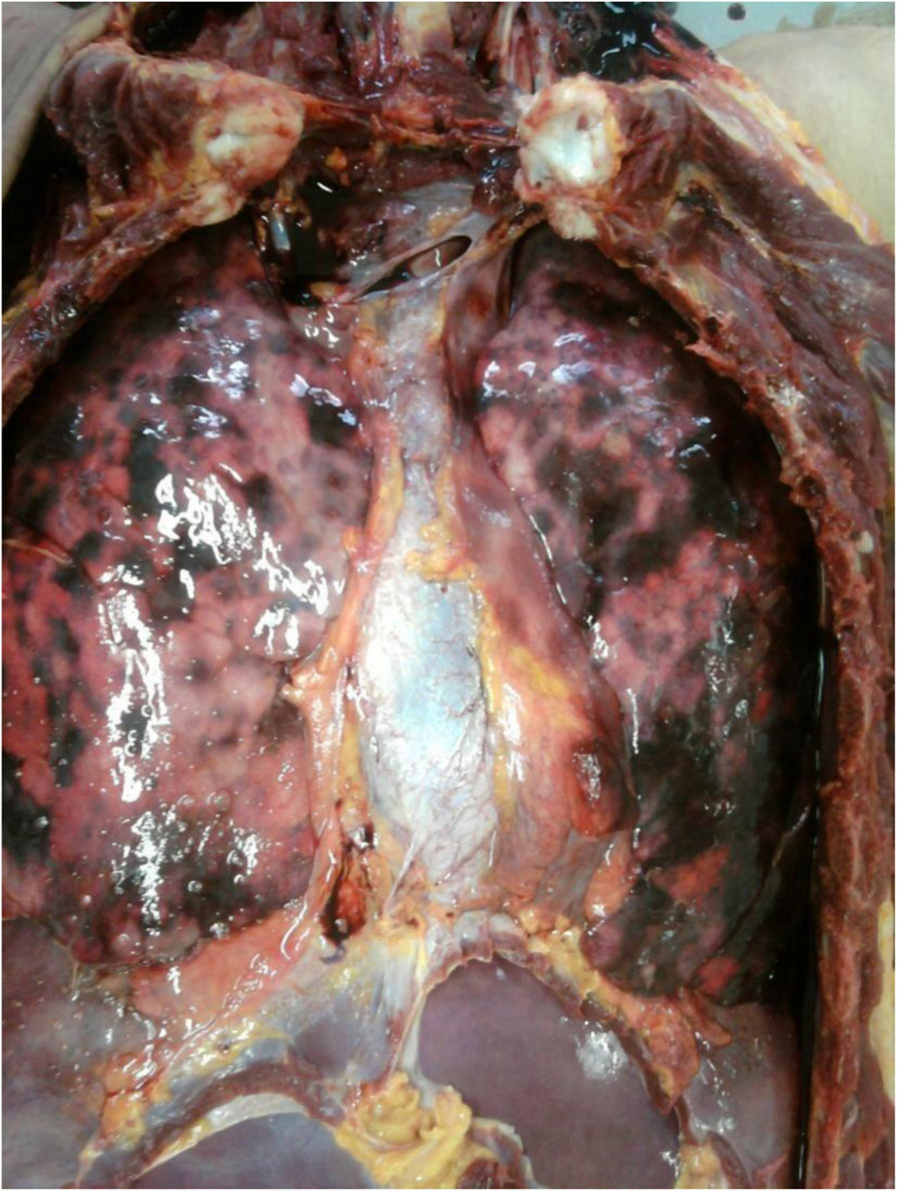
Diffuse haemorrhagic alveolitis related to severe bronchopulmonary infection

Sudden death of the young adult was due to pleural or pulmonary infection in four cases (Fig. 3), to an asthma attack, and to pneumothorax both in two cases. The abdominal cause of death was found in eight cases. It was, especially due to liver hydatid cyst rupture, peritonitis/intestinal occlusion, and pheochromocytoma, etc. … (Table 2). The rupture of a cerebral aneurysm was seen in two cases.

### Undetermined cause of death

In 13 cases, the autopsy, and the complementary examinations did not make it possible to determine the cause of death. In some cases, tissue lesions have been found but cannot by themselves explain death. Death remains indeterminate, especially in the age group between 18 and 24 years (46.2%). There is a clear male predominance: eight men, and five women with a sex ratio of 1.6. Sudden death in the family was not found. Regarding personal history, no antecedent was encountered.

The heart was macroscopically normal in 10 cases (76.9%). The myocardium was homogeneous in all cases. In the majority of cases, coronaries were free in eight cases (61.5%). There was in no case a significant obstruction of the coronaries. Pulmonary oedema was noted in 10 cases (76.9%). Hepatic congestion was described in eight cases (61.5%). Cerebral oedema was reported in 11 cases (84.6%). In all these cases the pathological examination failed to determine the cause of death.

## Discussion

In our study, we started this work to determine the features of sudden death in young adults in the study population and to determine the etiologies of sudden death in this age interval. In this series, sudden death in young adults occurs mainly due to cardiovascular diseases, especially related to myocardial ischemia in male subjects. In addition, sudden death was in the morning and early in the week. It was more common in summer. Sudden death is most often the first manifestation of pathologies, especially unsuspected heart diseases.

The predominance of cardiovascular causes is the common denominator of almost all studies reported in the literature. The understanding of the sudden death of the young adult is interesting to develop prevention strategies. Indeed, the etiologies are very variable. They differ according to the studied population (age, sex, ethnic origin, diet ...). A definition of sudden death was adopted by the World Health Organization [7]. In our study, the inclusion criteria used the most recognized definition of sudden death in general, which is that of the World Health Organization.

Sudden death of the young adult in itself was rarely studied as a separate entity. The majority of studies were limited to one age group (sudden death in infants ...) or a particular group of people (sudden death in athletes, sudden death in the military, sudden death instead of work ...) with the possible risk of selection bias.

The second problem that is encountered is the divergence in studies that define “young adult”. In our study, we saw the utility of grouping all subjects aged 18 to 35 years. The choice of the upper age limit at age 35 is identical to the majority of articles in the literature [2, 8, 9].

The incidence studies were divergent since the sample was not the same for inclusion ages. In a 10-year US College of Cardiology (2000–2010) study of subjects performing military service, the incidence of sudden death was 3.25/100,000 for subjects aged 18 to 35 years old [8]. This incidence was at 2.68 in Ireland [3]. There is indeed a divergence in the incidence. This wide divergence shows that the incidence of sudden death, including that of the young adult, is dependent on the specific geographical, ethnic, socioeconomic factors of each country [3].

In the current study, the average age was 26.4 years old. In general, sudden death spares no age range [1]. Nevertheless, two peak frequencies were noted in our study population at 21 (6.4%), and 35 (7.7%), respectively. In a Danish study, the peak frequency was at the age of 35 years old [2]. In a Danish nationwide cohort of persons aged < 50 years, the annual incidence rate of sudden cardiac death was of almost 10 times higher in persons aged 36 to 49 years than in persons aged 1 to 35 years [10].

The peaks already described could be explained by the hyperactivity of the population at these ages associated with a change in the habits of life of the subjects with mostly the consumption of alcohol, and tobacco and exposure to stress. These united factors favour decompensating a probable heart disease until there unknown. Indeed, the older the age, the more the risk of sudden death increases as risk factors accumulate [8].

In our study, the sex ratio was 3.6. In all published studies, there is still a male predominance of sudden death of the young subject. This predominance varies from one study to another, and is between 83 and 100% [11, 12]. In the Fingesture study, women were considerably older at the time of sudden death, and more commonly had non-ischemic causes. Women were also more likely to have a prior normal clinical finding than men, but an increased biological marker for sudden death risk criteria for left ventricular hypertrophy with repolarization abnormalities was more commonly observed in women [13]. In addition to these non-modifiable risk factors (sex, hormones, etc.), lifestyle also seems to be implicated in the male predominance of sudden death. Smoking, and alcohol use, affecting men more than women, appear to be factors contributing to this inequality [14].

In this work, a family history of sudden death was noted only in 7 cases (5.1%). Medical history of sudden death in the family has been variable in the literature between 1.38, and 5.26%. The presence of a family history of sudden death is contributing for some pathologies such as hypertrophic cardiomyopathy, arrhythmogenic dysplasia of the right ventricle, coronary malformations, and long QT syndrome [15].

In a prospective study in France, a significant correlation between the antecedent of sudden death in the family, and the risk of its occurrence in another member was found [16]. The FRAMINGHAM study [14] showed that the occurrence of coronary death in a parent increased by 30% the risk of coronary heart disease in children. How could these subjects survive asymptomatic until sudden death? Is there a problem in the interrogation? Whatever the problem, these findings lead us to recommend a better interview with the parents, although they are in such conditions still in shock.

Cardiovascular origin is at the top of the list of etiologies of sudden death in young adults. This predominance of cardiovascular causes of sudden death is the common denominator of all series reported in the literature, regardless of the demographic, and geographical characteristics of the study population. The cardiovascular causes of sudden death, in general, are different according to the age group. Indeed, in young people (< 35 years), it is the primary cardiomyopathies, and cardiac arrhythmias most frequently incriminated in sudden death [2].

In our study, ischemic heart disease is responsible for sudden death in 32.3% of cases. Ischemic heart disease in young adults takes on a particular aspect. Indeed, it is not necessarily coronary atherosclerosis at the origin of ischemic heart disease. In addition to “major” malformations, which are symptomatic from an early age, or sometimes in adolescents or young adults, certain “minor” abnormalities remain silent [17]. An American study on military subjects (age < 35 years) showed a probable cardiac origin in 41.3% of cases, coronary artery disease in 23.2% of cases, and hypertrophic cardiomyopathy in 12.8% of cases.

In the current study, in 9.5% of the cases, the autopsy, and the complementary investigations did not yield to make a certain diagnosis. The percentage of obscure autopsies or when the observed lesions do not sufficiently explain the death varies from one study to another. This variation in the percentage depends on the means of complementary examinations used. This percentage is also variable according to the age group. After reviewing the literature, the obscure autopsy is noted in 20, and 26% [2, 3].

This can be explained by the difference between the age groups studied. Death by inhibition is a suspicious death, given the more or less accidental nature of the inhibition that may be consecutive to trauma: boxing, judo, stroke, puncture, abortion, drowning [18]... The essential functional sudden death represents 1 to 17% according to the series [19].

In the current study, death occurred during physical activity in 15 cases. It is recognized that regular physical exercise requires long-term cardiovascular protection [20]. Death during physical activity often occurs during the recovery period [21]. In a British study, only 20% of hypertrophic cardiomyopathy patients were diagnosed during life, underscoring how often the disease is not identified during life, and sudden cardiac death can be the first manifestation. Moreover, the majority of sudden cardiac death events occurred at rest, and some even during sleep; the variables associated with death during exercise were young age, and male sex [22].

As in the present study, the summer predominance is comparable to that found in the literature [23, 24]. This slight predominance of cases of sudden death during the summer may be related to the excessive heat in our country at this time of year (from June to October). The seasonal variation in the incidence of sudden death in a Japanese population showed that it increased in agricultural workers in April, and September and employees in March, and September [25]. This could be explained by the stress encountered by each type of work. In addition, the Gulf region and Malaysia, are known to be hot countries, and daily workers does not have enough rest in Summer [24, 26]. A majority of them comprises labourers, factory workers, drivers and so on (30.6%). This group has lower income compared to other groups. Due to the financial problem, they might not get a regular medical check-up for early detection of their diseases or even treatment for their diseases [26].

The distribution of deaths by day of the week showed a peak frequency for Tuesday. This could be related to the return to work as well as stress submission after weekend rest. In our series, two frequency peaks during the day were noted at 7 am, and 8 am. Sudden death was more common between 4 am, and 11 am (48.7%). The concept of circadian variation in sudden death was also noted in the FRAMINGHAM study [14] with a risk of sudden cardiac death 3 times greater in the morning than in the evening.

In the current study, most deaths occurred at rest (or minimal daily activity). This result is consistent with the majority of studies published in the literature [8, 21, 22, 27]. This predominance could be explained by the fact that in the indeterminate forms of death, and hypertrophic cardiomyopathy, the death is rather the consequence of a rhythm disorder whose occurrence is often at rest or during sleep. Moreover, faintness is described as a premonitory sign in 59.1% of cases. There is a divergence in the frequencies described by the different studies. This can be explained by a terminology problem [8]. Indeed, the meaning of the word “faintness” is a bit vague for the forensic scientist as well as for the testimony.

At present, and especially in those cases where the classical autopsy is unable to determine a certain cause of death, we can no longer speak of a forensic autopsy in this context without resorting to genetic investigations. Indeed, deaths attributable to cardiac arrhythmia, dysfunction of the electrical system of the heart are often hereditary in nature. The diagnosis cannot be made during the traditional autopsy, because the cardiac tissue may be free of any visible sign, hence the need for a genetic autopsy [28]. The results of the autopsy were negative. Then, death is often attributable to an arrhythmia that leaves no identifiable sign in the heart tissue. Most of the disorders that affect the heart’s electrical system are related to an inherited genetic abnormality, which can be found in many members of the same family. Given the often-asymptomatic nature of these disorders, their first manifestation may be a fatal cardiac event. However, there is a recruitment bias since cases where a pathological examination was not performed, and when the forensic record did not have enough information were eliminated.

Among the limitations of our study, the genetic investigation focused on heart tissue was not performed. Molecular autopsy plays an important role in characterizing the existence of a certain genetic determinism of sudden death in young adults. There was sometimes a lack of data on the deceased’s medical history. In addition, this series does not cover all the cases of sudden death in the region of Monastir. In this situation, we cannot talk about epidemiological findings.

## Conclusion

To our knowledge, and after exhaustive research in the literature, our study is among the few studies on sudden death in the young subject, which takes into consideration all the causes of death. In our study, the objectives were to describe the characteristics of sudden death of the young subject, to specify the different causes of death.

In this series, sudden death in young adults occurs mainly in a smoking male, aged between 18 and 24 years old, occurring at rest, in the morning, and early in the week. It is more common, especially in summer. Sudden death is most often the first manifestation of pathologies, especially unsuspected heart diseases. The predominance of cardiovascular causes is the common denominator of almost all studies reported in the literature.

The review of the causes of sudden death of the young subject in our study showed that certain cardiovascular origins represent the first cause of death. Sudden death has remained undetermined in 9.5% cases. This important percentage is consistent with the literature. In these cases, death may be explained by a probable cardiac cause, the identification of which requires the performance of a molecular autopsy. However, sudden death may remain of indeterminate cause. The identification of subjects at risk of sudden death remains a hope, and a goal to be achieved. This will help take preventive measures for the rest of the family.

## Data Availability

The datasets generated and/or analysed during the current study are available from the corresponding author on reasonable request.

## References

[1] Fontaine A, Barraine P, Pavic C, Parent P, Marcorelles P, L’Her E (2013). Le médecin légiste et la mort subite du sujet jeune. Rev Méd Légale.

[2] Winkel BG, Holst AG, Theilade J, Kristensen IB, Thomsen JL, Ottesen GL (2011). Nationwide study of sudden cardiac death in persons aged 1-35 years. Eur Heart J.

[3] Margey R, Roy A, Tobin S, O’Keane CJ, McGorrian C, Morris V (2011). Sudden cardiac death in 14- to 35-year olds in Ireland from 2005 to 2007: a retrospective registry. EP Eur.

[4] Risgaard B (2016). Sudden cardiac death: a nationwide cohort study among the young. Dan Med J.

[5] Durante A, Laforgia PL, Aurelio A, Foglia-Manzillo G, Bronzato S, Santarone M (2015). Sudden cardiac death in the young: the bogeyman. Cardiol Young.

[6] Chaturvedi M, Satoskar M, Khare M, Kalgutkar A (2011). Sudden, unexpected and natural death in young adults of age between 18 and 35 years: a clinicopathological study. Indian J Pathol Microbiol.

[7] Organization WH (1993). International classification of diseases (ICD-10).

[8] Eckart RE, Shry EA, Burke AP, McNear JA, Appel DA, Castillo-Rojas LM (2011). Sudden death in young adults: an autopsy-based series of a population undergoing active surveillance. J Am Coll Cardiol.

[9] Chandra N, Bastiaenen R, Papadakis M, Sharma S (2013). Sudden cardiac death in young athletes: practical challenges and diagnostic dilemmas. J Am Coll Cardiol.

[10] Burden of sudden cardiac death in persons aged 1 to 49 years | Circ Arrhythm Electrophysiol. 10.1161/CIRCEP.113.001421. 10.1161/CIRCEP.113.001421 24604905

[11] Drory Y, Turetz Y, Hiss Y, Lev B, Fisman EZ, Pines A (1991). Sudden unexpected death in persons <40 years of age. Am J Cardiol.

[12] Phillips M, Robinowitz M, Higgins JR, Boran KJ, Reed T, Virmani R (1986). Sudden cardiac death in air force recruits: a 20-year review. JAMA..

[13] Haukilahti MAE, Holmström L, Vähätalo J, Kenttä T, Tikkanen J, Pakanen L (2019). Sudden cardiac death in women: causes of death, autopsy findings, and electrocardiographic risk markers. Circulation..

[14] Kannel WB, Thomas HE (1982). Sudden coronary death: the Framingham study*. Ann N Y Acad Sci.

[15] Doolan A, Semsarian C, Langlois N (2004). Causes of sudden cardiac death in young Australians. Med J Aust.

[16] Empana J-P, Ducimetière P, Jouven X (2008). Facteurs de risque de mort subite de l’adulte en population générale. MT Cardio.

[17] Kealey AJ (2010). Coronary artery disease and myocardial infarction in pregnancy: a review of epidemiology, diagnosis, and medical and surgical management. Can J Cardiol.

[18] Tikkanen JT, Viktor W, Juhani JM, Meri R, Eeva H, Olli-Pekka L (2012). Association of early repolarization and sudden cardiac death during an acute coronary event. Circ Arrhythm Electrophysiol.

[19] Viskin S, Rosso R, Halkin A (2012). Explaining sudden unexplained death. Circ Arrhythm Electrophysiol.

[20] Dahabreh IJ, Paulus JK (2011). Association of episodic physical and sexual activity with triggering of acute cardiac events: systematic review and meta-analysis. JAMA..

[21] Allouche M, Boudriga N, Ahmed HB, Banasr A, Shimi M, Gloulou F (2013). La mort subite au cours d’une activité sportive en Tunisie : à propos d’une série autopsique de 32 cas. Ann Cardiol Angeiol.

[22] Finocchiaro G, Papadakis M, Sharma S, Sheppard M (2017). Sudden cardiac death. Eur Heart J.

[23] Mesrati MA, Belhadj M, Aissaoui A, HajSalem N, Oualha D, Boughattas M (2017). La mort subite cardiovasculaire de l’adulte : étude autopsique de 361 cas. Ann Cardiol Angeiol.

[24] Nofal HK, Abdulmohsen MF, Khamis AH (2011). Incidence and causes of sudden death in a university hospital in eastern Saudi Arabia. East Mediterr Health J.

[25] Hayashi S, Toyoshima H, Sato T, Tanabe N, Seki N, Miyanishi K (1997). Seasonal variation in the incidence of sudden death according to occupation of householder in Japan. Jpn Circ J.

[26] Kumar V, San KP, Idwan A, Shah N, Hajar S, Norkahfi M (2007). A study of sudden natural deaths in medico legal autopsies in University Malaya Medical Centre (UMMC), Kuala Lumpur. J Forensic Legal Med.

[27] Corrado D, Basso C, Rizzoli G, Schiavon M, Thiene G (2003). Does sports activity enhance the risk of sudden death in adolescents and young adults?. J Am Coll Cardiol.

[28] Loire R, Tabib A. [Unexpected sudden cardiac death. An evaluation of 1000 autopsies]. Arch Mal Coeur Vaiss 1996;89(1):13–8. http://europepmc.org/abstract/MED/8678733. 8678733

